# The cGAS-STING pathway in metabolic dysfunction-associated steatotic liver disease: mechanistic insights and therapeutic opportunities

**DOI:** 10.3389/fphar.2026.1909062

**Published:** 2026-10-13

**Authors:** Xingxing Yuan, Jing Li, Ying Li, Lei Yang

**Affiliations:** 1 Department of Gastroenterology, Heilongjiang Academy of Traditional Chinese Medicine, Harbin, China; 2 Department of Graduate Studies, Academy of Traditional Chinese Medicine, Harbin, China; 3 Department of Nephrology, Heilongjiang Academy of Traditional Chinese Medicine, Harbin, China

**Keywords:** cGAS-STING pathway, innate immunity, liver fibrosis, metabolic dysfunction-associated steatotic liver disease, targeted therapy

## Abstract

Metabolic dysfunction-associated steatotic liver disease (MASLD) is a highly prevalent chronic metabolic liver disease worldwide, encompassing a spectrum that includes simple steatosis, metabolic dysfunction-associated steatohepatitis (MASH), liver fibrosis, and hepatocellular carcinoma (HCC), with immune-metabolic-inflammatory dysregulation at its core pathogenesis. The cyclic GMP-AMP synthase-stimulator of interferon genes (cGAS-STING) pathway serves as the central axis of cytosolic DNA sensing and innate immune activation. Endogenous stimuli, including mitochondrial DNA leakage, DNA damage, and gut microbial DNA translocation, can persistently activate this pathway, which in turn regulates lipid metabolism imbalance, hepatic inflammation amplification, hepatic stellate cell (HSC) activation, and malignant transformation through downstream signaling axes, driving the full spectrum of MASLD pathology. Molecules associated with the cGAS-STING pathway may serve as non-invasive diagnostic biomarkers for MASLD, and natural compounds, traditional Chinese medicine, and nanosystems targeting this pathway have demonstrated promising therapeutic effects. This review systematically summarizes the molecular mechanisms of the cGAS-STING pathway, its regulatory roles in MASLD, and advances in targeted therapy, providing a theoretical foundation for disease mechanism elucidation and the development of diagnostic and therapeutic strategies.

## 1 Introduction

Metabolic dysfunction-associated steatotic liver disease (MASLD), formerly termed non-alcoholic fatty liver disease (NAFLD), is a chronic progressive liver disease driven by overnutrition and insulin resistance in genetically susceptible individuals (Ren et al., 2026). The nomenclature was updated in 2023 by a multi-society Delphi consensus to better reflect the metabolic basis of the disease, with the diagnostic criterion requiring the presence of at least one cardiometabolic risk factor (Tacke et al., 2024). MASLD represents the full disease spectrum, which begins with metabolic dysfunction-associated steatotic liver (MASL)—the early stage defined by isolated hepatic steatosis without significant inflammation or hepatocellular injury—and progresses to metabolic dysfunction-associated steatohepatitis (MASH), and can further advance to cirrhosis and hepatocellular carcinoma (HCC) (Rinella et al., 2023). A systematic review and meta-analysis encompassing over 1.03 million participants from 17 countries revealed a global MASLD prevalence of 32.4%, with a continuously rising trend; the prevalence was significantly higher in males (39.7%) than in females (25.6%), with an overall incidence of 46.9 per 1,000 person-years (Riazi et al., 2022). Critically, MASLD has become the leading contributor to the rapidly increasing incidence of HCC in developed countries including the United States, France, and the United Kingdom. The overall incidence of MASLD-related HCC is 1.25 per 1,000 person-years, which escalates to 14.46 per 1,000 person-years in patients with advanced fibrosis, representing a substantial disease burden (Huang et al., 2021; Thomas et al., 2022). At present, the core pathogenic mechanisms of MASLD remain incompletely understood, and safe and effective pharmacological interventions and therapeutic targets are still lacking in clinical practice. Elucidating its pathogenesis is therefore essential for improving disease diagnosis and treatment.

The cyclic GMP-AMP synthase (cGAS)-stimulator of interferon genes (STING) signaling pathway is the central signaling axis for cytosolic DNA sensing and innate immune activation in mammals (Yu and Liu, 2021). When double-stranded DNA (dsDNA) from pathogens or host cellular damage appears in the cytosol, cGAS specifically recognizes and binds dsDNA, catalyzing adenosine triphosphate (ATP) and guanosine triphosphate (GTP) to generate the second messenger 2′3′-cyclic GMP-AMP (2′3′-cGAMP). This second messenger subsequently binds to and activates STING on the endoplasmic reticulum (ER) membrane, initiating downstream signaling cascades that induce the expression of type I interferons and pro-inflammatory cytokines, mediating innate immune and chronic inflammatory responses (Pan et al., 2023).

Accumulating evidence has delineated the multilayered regulatory mechanisms of this pathway at the post-translational and non-coding RNA levels, and highlighted its conserved role as a core immunometabolic hub across the spectrum of chronic liver diseases, including MASLD, autoimmune liver diseases, and viral hepatitis (Xu et al., 2023; Wang Y. et al., 2025). Numerous studies have confirmed that the cGAS-STING pathway not only plays a critical role in anti-infective immunity but is also widely involved in the pathological processes of tumors, autoimmune diseases, and neurodegenerative diseases (Liu and Pu, 2023; Wang W. et al., 2025; Rathod et al., 2026). Recent evidence further indicates that cGAS-STING plays an important role in metabolic disorder-associated inflammatory diseases, particularly in the development, progression, and malignant transformation of MASLD. Mitochondrial DNA (mtDNA) released from damaged mitochondria serves as the core endogenous ligand that activates this pathway, driving disease progression through multiple mechanisms including oxidative stress, inflammatory amplification, and programmed cell death (Gong et al., 2024; Li et al., 2024).

Collectively, emerging evidence confirms that endogenous stimuli including mitochondrial DNA leakage, nuclear DNA damage, and gut microbial DNA translocation induce persistent hepatic cGAS-STING overactivation. As a core immune-metabolic regulatory hub, this pathway activates canonical and non-canonical downstream signaling axes, exerting cell-type-specific and stage-dependent bidirectional effects, both homeostatic/protective and pathogenic, across the full spectrum of MASLD progression, from steatosis and MASH inflammation to fibrosis and HCC transformation. Nevertheless, current reviews lack systematic integration of its sophisticated regulatory mechanisms, bidirectional stage-specific functions, biomarker potential, and novel targeted therapies, particularly updated advances in natural compounds, traditional Chinese medicine, exosomes, and nano-delivery systems. Accordingly, this review systematically summarizes the cGAS-STING molecular network and its stage-specific roles in MASLD, updating cutting-edge diagnostic and therapeutic progress to provide a theoretical basis for precise clinical intervention for MASLD.

## 2 Molecular mechanisms of the cGAS-STING signaling pathway

Discovered in 2013, the cGAS-STING pathway represents a core frontier of innate immune research responsible for sensing abnormal cytosolic DNA (Yu and Liu, 2021). Its activity is tightly regulated by post-translational modifications, non-coding RNAs, and subcellular compartmentalization, and dysregulation of this homeostatic control is a common driver of chronic inflammatory and metabolic diseases (Wang Y. et al., 2025). This section elaborates the canonical signal transduction cascade, alongside succinct supplementary explanations of non-canonical signaling branches and homeostatic negative regulatory mechanisms of this universal DNA-sensing pathway.

### 2.1 Canonical activation of the cGAS-STING pathway

#### 2.1.1 DNA recognition by cGAS and second messenger synthesis

cGAS, encoded by the *MB21D1* gene and classified as a nucleotidyltransferase family member, serves as the upstream DNA sensor that initiates pathway activation (Michalski et al., 2020). The protein contains an N-terminal positively charged flexible domain and a C-terminal catalytic domain equipped with zinc finger motifs that mediate sequence-independent binding to dsDNA; dsDNA fragments longer than 20 bp are the minimal threshold to trigger cGAS activation (Jiang Z. et al., 2025). Under resting conditions, cGAS exists as an inactive monomer with a closed catalytic pocket. Once bound to aberrant cytoplasmic dsDNA—including pathogen-derived DNA, leaked mitochondrial DNA and damaged nuclear chromatin fragments—cGAS undergoes conformational rearrangement to form a 2:2 cGAS–dsDNA complex, which catalyzes ATP and GTP to synthesize the second messenger 2′3′-cGAMP (Li and Su, 2020).

The distinct subcellular localization of cGAS establishes a self/non-self DNA discrimination system to prevent spontaneous inflammatory activation (Gekara and Jiang, 2019). Approximately 70%–80% of endogenous cGAS resides in the nucleus to monitor genomic DNA damage and micronucleus formation, whereas cytoplasmic cGAS mainly detects exogenous pathogenic DNA and endogenously leaked nucleic acids to trigger innate immune responses (Zhao et al., 2020; Lu et al., 2024). The N-terminal phosphoinositide-binding domain anchors cytoplasmic cGAS to the inner plasma membrane, reducing non-specific binding to irrelevant intracellular nucleic acids and improving recognition specificity (Barnett et al., 2019).

#### 2.1.2 STING activation and downstream kinase cascade

STING (also named TMEM173), encoded by the *TMEM173* gene, is an evolutionarily conserved four-pass transmembrane protein localized on the ER membrane under basal conditions (Zhang et al., 2021). Binding of 2′3′-cGAMP induces drastic conformational changes in STING monomers to form active dimers and linear oligomers, which subsequently translocate from the ER to the Golgi apparatus via vesicular trafficking; this ER-to-Golgi transport is an indispensable prerequisite for downstream signal transduction (Taguchi et al., 2021; Yu et al., 2022). The C-terminal cytoplasmic domain of STING harbors a conserved pLxIS motif, which functions as the core platform to recruit downstream signaling kinases (Hussain et al., 2022).

Oligomerized STING at the Golgi apparatus recruits TBK1 through the pLxIS motif, triggering TBK1 autophosphorylation (Yu et al., 2019). Activated TBK1 further phosphorylates IRF3, and phosphorylated IRF3 homodimerizes and translocates into the nucleus to initiate transcription of type I interferons and interferon-stimulated genes (Yum et al., 2021). Meanwhile, STING also activates the IKK complex to accelerate proteasomal degradation of IκB, releasing NF-κB to enter the nucleus and drive robust expression of pro-inflammatory cytokines including TNF-α, IL-1β and IL-6 (Balka et al., 2020). Figure 1 **.**

**FIGURE 1:**
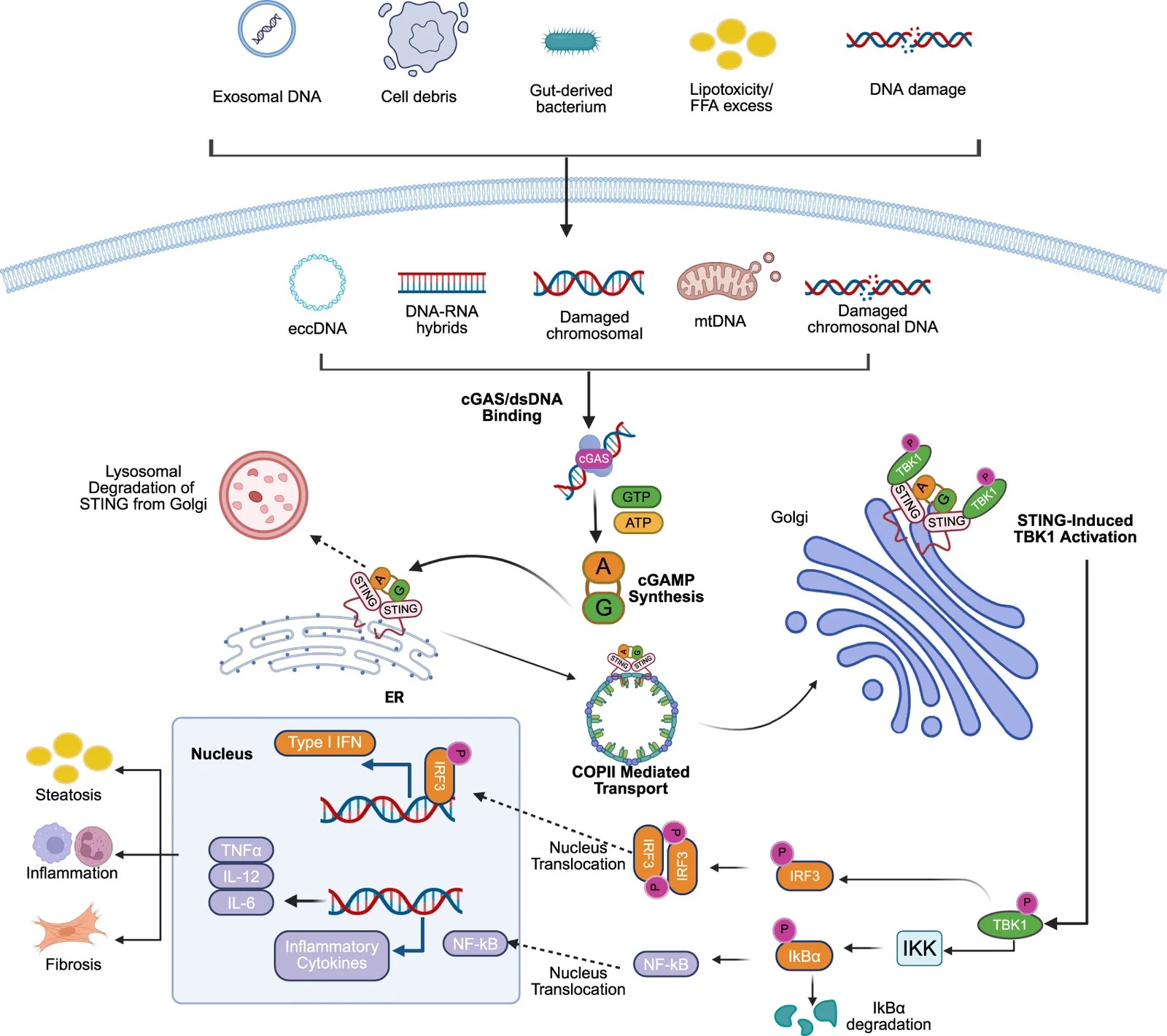
Canonical cGAS-STING signaling pathway.

### 2.2 Non-canonical signaling branches and homeostatic negative regulation

Beyond canonical interferon and pro-inflammatory cytokine production, the cGAS-STING pathway engages two representative non-canonical signaling cascades. The cGAS-STING-NLRP3 inflammasome axis drives caspase-1/GSDMD activation to induce pyroptosis and exacerbate local inflammatory tissue injury (Chen K. Q. et al., 2025). Meanwhile, STING directly recruits autophagy-related proteins WIPI2 and ATG5 to launch interferon-independent autophagy, which eliminates damaged organelles and cytoplasmic dsDNA to sustain cellular homeostasis (Wan et al., 2023). Notably, this autophagic function displays cell-type specificity: cGAS facilitates STING-independent autophagy in parenchymal cells, whereas STING primarily governs inflammatory and pyroptotic responses in myeloid cells (Luo et al., 2023).

Physiologically, multiple layered negative regulatory circuits constrain sustained STING hyperactivation to prevent aberrant, overwhelming tissue inflammation. Once signal transduction finishes, activated STING undergoes Rab7a-dependent autophagic delivery to lysosomes for degradation, which rapidly ceases inflammatory signal transmission (Sun and Hornung, 2022). Additionally, ROS mediate oxidative modification of conserved cysteine residues on STING, disrupting STING oligomerization and TBK1 recruitment to restrain excessive pathway activation (Zamorano Cuervo et al., 2021). At the upstream sensing level, caspase-1 and caspase-3 directly proteolyze cGAS to disrupt its DNA-binding and catalytic domains, thereby blocking pathway initiation at the source (Xiong et al., 2021).

## 3 The cGAS-STING signaling pathway in MASLD

Aberrant activation of the cGAS-STING pathway is a core immune-metabolic driver of MASLD progression. Notably, the pathway exerts stage-dependent bidirectional effects: moderate physiological activation mediates homeostatic and tissue-protective responses, while sustained aberrant overactivation drives pathological progression. This bidirectional regulation is further shaped by cell-type-specific functions across hepatic parenchymal, immune, and stromal cells. This pathway senses mtDNA released from injured hepatocytes, nuclear DNA damage fragments, and microbial DNA leaking from the gut, thereby activating downstream signaling axes including TBK1-IRF3, NF-κB, and mTORC1. It participates throughout the disease process from hepatic steatosis, inflammatory infiltration, and fibrosis formation to malignant transformation, with regulatory effects exhibiting notable cell-type and pathological stage specificity, positioning it as a key hub connecting metabolic dysregulation, innate immunity, and liver injury.

### 3.1 Regulation of hepatic steatosis: initiation of early MASL pathology

The cGAS-STING pathway directly drives aberrant hepatic lipid accumulation and initiates early MASL pathological changes by disrupting lipid metabolism balance and suppressing lipophagy. Mitochondrial quality control (MQC) impairment—characterized by defective mitophagy, imbalanced mitochondrial fission-fusion, and impaired mitochondrial biogenesis—has been identified as a central upstream driver of MASLD pathogenesis; this MQC dysfunction is a major source of leaked mtDNA that sustains cGAS-STING pathway overactivation under lipotoxic stress (Fu et al., 2026). Under lipotoxic stimulation, hepatocyte mitochondrial damage releases mtDNA, which activates the cGAS-STING pathway. This activation stimulates mTORC1 via SQSTM1, significantly suppressing hepatic lipophagy, leading to impaired lipid droplet degradation and substantial triglyceride accumulation. Concurrent STING and mTORC1 activation have been detected in liver tissues of MASLD patients, further confirming the central role of this pathway in lipid accumulation (Liu et al., 2022). Meanwhile, aberrantly high expression of SREBP cleavage-activating protein (SCAP) in macrophages recruits the STING-TBK1 complex for Golgi translocation, activating the STING-NF-κB signaling axis and promoting pro-inflammatory cytokine release, which in turn enhances adipocyte lipolysis and hepatocyte lipid uptake, driving ectopic lipid deposition in lean MASLD (Huang et al., 2022). High-fat diet-induced hepatocyte nucleotide pool imbalance triggers replication stress and DNA damage, which can also persistently activate the cGAS-STING pathway, coupling metabolic stress with innate immune activation and initiating early hepatic inflammatory responses, thereby laying the groundwork for the progression from steatosis to inflammation (Donne et al., 2022).

Beyond these established mechanisms, the macrophage STING-YAP axis has emerged as a critical regulator of lipid metabolism in preclinical models. STING activation in macrophages promotes YAP activity, which in turn facilitates the autophagic degradation of lipid droplets. This counterintuitive phenotype is explained by a negative feedback loop: STING activation concurrently restrains excessive YAP activity via NF-κB signaling; STING deletion removes this restraint and causes YAP hyperactivation and reduced lipid accumulation, whereas STING/YAP double knockout abolishes YAP-dependent lipophagy and exacerbates hepatic lipid deposition (Yang et al., 2024).

In hepatocytes, the STING-IRF3 pathway directly promotes cellular inflammation and apoptosis while concurrently inhibiting glycolysis and lipolysis and promoting gluconeogenesis and lipogenesis, thereby actively driving metabolic dysregulation in MASLD (Qiao et al., 2018). Thus, the cGAS-STING pathway drives hepatic steatosis through at least two complementary mechanisms: hepatocyte-intrinsic metabolic reprogramming via the STING-IRF3 axis and macrophage-mediated lipid regulatory crosstalk via the STING-YAP pathway.

Of note, at the early steatosis stage, basal cGAS-STING signaling also exerts homeostatic protective effects: it promotes autophagic clearance of damaged mitochondria and excess lipid droplets in hepatocytes, and maintains genomic stability in the nucleus to limit persistent pathway overactivation (Zhao et al., 2020; Wan et al., 2023).

### 3.2 Mediation of hepatic inflammation amplification: promoting MASL-to-MASH transition

When lipid accumulation exceeds homeostatic thresholds, the cGAS-STING pathway becomes a core mediator of MASH inflammatory amplification, exacerbating hepatocyte injury and inflammatory infiltration through multi-cellular interactions. STING expression is significantly upregulated in Kupffer cells and monocyte-derived macrophages, where it activates NF-κB signaling as the core mtDNA-sensing receptor, leading to abundant release of pro-inflammatory cytokines including TNF-α, IL-1β, and IL-6, directly inducing hepatocyte ballooning degeneration, apoptosis, and necrosis (Luo et al., 2018; Yu et al., 2019; Wang et al., 2020). Simultaneously, STING can trigger caspase-1/GSDMD-mediated hepatocyte pyroptosis by activating the NLRP3 inflammasome, further amplifying local hepatic inflammation. Saturated fatty acid stimulation activates the cGAS-STING-TBK1 axis, mediating p62 phosphorylation and promoting the formation of ubiquitinated protein aggregates, which serve as key pathological hallmarks distinguishing simple steatosis from MASH (Cho et al., 2018). In parallel, the Foxo1-YAP-Notch1 axis in macrophages can reprogram STING-mediated innate immune responses. High-fat diet-induced oxidative stress concurrently activates these signaling molecules, exacerbating hepatic inflammation and steatosis (Xu et al., 2024). Moreover, glycolysis and the STING pathway form a positive feedback loop: glycolytic products (ATP) promote STING activation, and activated STING reciprocally enhances glycolysis, maintaining the M1 pro-inflammatory macrophage phenotype and continuously driving the MASL-to-MASH transition (Chen P. et al., 2025).

The gut–liver axis also confers context-dependent bidirectional effects on MASH progression (de Carvalho Ribeiro et al., 2023). In intact intestinal epithelium, physiological cGAS-STING signaling maintains tight junction integrity and prevents microbial DNA translocation, exerting a protective effect; when the intestinal barrier is disrupted, translocated microbial DNA activates hepatic cGAS-STING and drives excessive inflammation and fibrosis.

### 3.3 Driving hepatic stellate cell activation and accelerating liver fibrosis

Under persistent stimulation by the chronic inflammatory microenvironment, the cGAS-STING pathway becomes a key driver of liver fibrosis progression by regulating the hepatocyte-macrophage-HSC crosstalk. mtDNA released from injured hepatocytes activates the cGAS-STING pathway in macrophages, promoting their polarization toward a pro-inflammatory phenotype and releasing pro-fibrotic factors such as TGF-β1, which in turn activates HSCs and induces extensive extracellular matrix deposition. The transcription factor TFAP4 can directly bind to the STING promoter region, transcriptionally activating the STING-TBK1-IRF3/NF-κB signaling axis and upregulating collagen fibers and pro-inflammatory cytokine expression, further amplifying the liver fibrosis process (Wang et al., 2020; Han et al., 2025). Nuclear receptor subfamily 1 group D member 1 (NR1D1) deficiency in HSCs leads to mitochondrial damage and mtDNA leakage, activating the cGAS-STING pathway and creating a pro-inflammatory microenvironment that directly accelerates fibrosis progression (Chen et al., 2023). Metabolic abnormalities such as iron overload and cholesterol metabolism disorders can also trigger hepatocyte pyroptosis and HSC activation by activating the cGAS-STING-NLRP3 axis (Li et al., 2022).

Notably, STING exhibits stage-specific bidirectional functions during liver fibrosis (Amer et al., 2025). In early-stage fibrosis, moderate STING activation enhances NK cell cytotoxic activity and promotes clearance of activated HSCs and damaged hepatocytes, exerting anti-fibrotic protective effects. In late-stage fibrosis, persistent STING overactivation drives chronic macrophage inflammation and HSC hyperactivation, while progressive downregulation of STING in immune cells impairs NK cell cytotoxic function, collectively driving sustained fibrosis progression.

### 3.4 Involvement in malignant transformation: mediating MASLD-associated HCC

With unresolved chronic inflammation and persistent fibrosis, sustained aberrant activation of the cGAS-STING pathway mediates the malignant transformation from MASLD to hepatocellular carcinoma (HCC) through immune microenvironment remodeling and regulation of cellular metabolic reprogramming. The E3 ubiquitin ligase RNF13 can degrade STING protein through ubiquitination, suppressing MASLD pathological progression; conversely, RNF13 deficiency leads to persistent STING activation in the liver, exacerbating insulin resistance, steatosis, inflammation, and fibrosis, and significantly increasing the risk of HCC (Lin et al., 2023). Sustained STING pathway activation suppresses the activation and infiltration of hepatic CD8^+^ cytotoxic T lymphocytes, mediating tumor immune evasion, while also regulating lipid metabolic reprogramming to provide energy support for HCC cell proliferation, driving the progression from liver fibrosis to HCC (Zhang et al., 2022).

Genomic instability and DNA damage are established upstream triggers of cGAS-STING pathway activation in hepatocytes. Conversely, sustained cGAS-STING activation amplifies DNA damage and genomic instability via chronic inflammatory stress and excessive ROS production, forming a pathogenic positive feedback loop that further accelerates hepatocyte malignant transformation (Gong et al., 2024; Chen K. Q. et al., 2025). This bidirectional interaction represents an important molecular mechanism of MASLD-associated HCC and provides potential targets for early HCC warning and targeted intervention.

Notably, the pathway also exerts anti-tumor protective effects in early pre-malignant stages, where STING activation enhances antigen cross-presentation and anti-tumor immune surveillance (Gong et al., 2024). The pathogenic effect dominates only when chronic inflammation drives persistent, aberrant pathway activation.

In addition to its direct pathogenic roles, the cGAS-STING pathway holds promise as a source of non-invasive biomarkers for MASLD. A circulating RNA panel centered on STING pathway-related molecules, including EDN1, TNF, MAPK3, and EP300, has been shown to be significantly upregulated in the serum of MASLD/MASH patients and correlates with fibrosis stage, offering potential utility for non-invasive MASLD diagnosis and fibrosis stratification (Albadawy et al., 2021). This translational dimension underscores the value of the cGAS-STING pathway as both a therapeutic target and a diagnostic tool in the clinical management of MASLD.

Collectively, the bidirectional functions of the cGAS-STING pathway are jointly determined by disease stage, activation intensity and cell type: protective homeostatic effects predominate at early disease stages with moderate activation, while sustained aberrant overactivation drives pathological progression across steatosis, MASH, fibrosis and HCC. This stage-dependent paradigm has critical implications for therapeutic strategy: pathway inhibition may be beneficial in advanced inflammation and fibrosis, but may impair homeostatic tissue defense and anti-tumor surveillance in early-stage disease.

## 4 Therapeutic strategies targeting the cGAS-STING signaling pathway

### 4.1 Inhibition of cGAS-STING pathway activation

Direct inhibition of the cGAS-STING pathway and its downstream TBK1-IRF3/NF-κB signaling axis represents a straightforward anti-inflammatory strategy for MASLD/MASH intervention in preclinical settings. Berberine ameliorates high-fat diet-induced hepatic steatosis, liver inflammation, and glucose metabolic disorders through concurrent activation of PI3K/Akt signaling and suppression of STING in mouse models and HepG2 cell assays (Ji et al., 2025). Targeting STING directly, paeoniflorin inhibits NLRP3 inflammasome-mediated hepatocyte pyroptosis and reduces lipid deposition, oxidative stress, and inflammatory injury in HFD-induced MASLD mice and AML-12 hepatocytes (Guo et al., 2025). In MASH mouse and cell models, 2H-ginsenoside Rg5 suppresses STING-TBK1-IRF3 pathway activation, attenuating type I interferon and pro-inflammatory cytokine release and improving both hepatic inflammation and fibrosis (Ma et al., 2025). Remdesivir blocks STING activity and downstream IRF3/NF-κB signaling, thereby reversing lipotoxicity-induced hepatocyte metabolic dysregulation and inflammatory responses in HFD-induced MASLD mice and PA-treated hepatocytes (Li and Su, 2020). By targeting the core nodes of the pathway, these small molecules rapidly control excessive hepatic inflammation in preclinical settings.

### 4.2 Inhibition of upstream mtDNA release and DNA damage

Reducing cytosolic dsDNA leakage at the source represents an upstream intervention strategy to block cGAS-STING pathway activation. By regulating the SHP-2/JAK2/BAX signaling axis, phloretin inhibits aberrant opening of the mitochondrial permeability transition pore and prevents mtDNA release, thereby suppressing cGAS-STING activation by cytosolic DNA and improving hepatic lipid metabolism and inflammatory responses in MASH mouse and cell models (Dong et al., 2025). Acting on liver sinusoidal endothelial cells, chlorogenic acid indirectly attenuates cGAS-STING-mediated inflammatory cell infiltration through the TGFβR1-ID1 signaling pathway, alleviating hepatic inflammatory injury in HFD/MCD-induced MASH mice (Ouyang et al., 2025). Soy isoflavones downregulate cGAS and STING expression via ERα-mediated signaling, curbing pathway overactivation and reducing hepatic fat accumulation while improving liver function in a gerbil MASLD model (Yang et al., 2025). These interventions exhibit favorable safety profiles in rodent studies, supporting further preclinical exploration for early MASL intervention.

### 4.3 Regulation of inflammatory microenvironment

Macrophages are the primary cellular vehicle through which the cGAS-STING pathway drives inflammation; modulating their polarization and function can effectively break the hepatic inflammatory loop in preclinical models. ErTao decoction and its core component naringin reduce hepatic collagen deposition and fibrotic lesions by suppressing STING-dependent macrophage activation and NLRP3 inflammasome activation in CCl_4_-induced mouse liver fibrosis models and cell models (Jia et al., 2025). Honokiol exerts a multi-pronged effect, concurrently blocking NLRP3 inflammasome activation, hepatocyte pyroptosis, and the cGAS-STING pathway, thereby suppressing the macrophage pro-inflammatory phenotype and alleviating cholesterol-induced liver injury and fibrosis in mice (Wang Z. et al., 2025). In MASH mouse models, human umbilical cord mesenchymal stem cell-derived exosomes, rich in miR-24-3p, target and suppress STING expression in macrophages, promoting polarization toward the anti-inflammatory M2 phenotype and significantly ameliorating hepatic steatosis, inflammation, and fibrosis (Jiang W. et al., 2025).

### 4.4 Induction of hepatic stellate cell senescence and degradation

Inducing senescence in activated HSCs and promoting their clearance is an emerging strategy for reversing liver fibrosis, and this process is highly dependent on the cGAS-STING pathway in preclinical settings. Ligustilide binds to G-actin, disrupting cytoskeletal and nuclear membrane integrity; the consequent dsDNA release activates the cGAS-STING pathway, driving HSC senescence and anti-fibrotic effects in mouse liver fibrosis models and activated HSC cultures (Qu et al., 2025). Oroxylin A acts via a distinct mechanism: it inhibits cGAS gene promoter methylation to activate cGAS-STING signaling, which in turn promotes ferritinophagy and induces HSC senescence, significantly alleviating liver fibrosis in mouse models (Sun et al., 2023; Zhao et al., 2023). Atractylenolide III, by contrast, suppresses the senescence-associated secretory phenotype through inhibition of the cGAS/NF-κB pathway, thereby reducing the pro-fibrotic activity of HSCs in etoposide-induced senescent human HSC models (Wu et al., 2023).

### 4.5 Targeted delivery and novel biologics

Targeted delivery systems and novel biologics can significantly enhance intervention specificity and safety in preclinical settings. The manganese-albumin nanosystem utilizes albumin-mediated transcytosis to precisely deliver manganese ions to activated HSCs, moderately activating the cGAS-STING pathway to induce cellular senescence and enhance immune clearance, thereby efficiently inhibiting liver fibrosis progression in mouse models (Gu et al., 2024). This system achieves specific targeting of diseased cells while avoiding non-specific effects on normal hepatocytes and immune cells, improving intervention safety in preclinical evaluations Table 1 **.**

**TABLE 1:** Preclinical therapeutic strategies targeting the cGAS-STING pathway for MASLD.

Compound	Experimental model	Mechanism	Efficacy metrics	References
Berberine	o *In vivo*: HFD-induced MASLD mouse model. *In vitro*: Human HepG2 cells	o Activates PI3K/Akt pathway and inhibits STING pathway	o Improves hepatic steatosis, inflammation, and glucose intolerance	(Ji et al., 2025)
Paeoniflorin	o *In vivo*: MASLD model induced by 16-week HFD in mice o *In vitro*: Mouse AML-12 hepatocytes	o Targets STING, inhibiting NLRP3 inflammasome-mediated pyroptosis	o Attenuates steatosis, oxidative stress, and inflammation	(Guo et al., 2025)
2H-ginsenoside Rg5	o *In vivo*: MASH mouse model o *In vitro*: MASH cell model	o Inhibits STING-TBK1-IRF3 pathway, reducing type I interferon release and inflammation	o Ameliorates lipid accumulation, inflammation, and fibrosis in MASH mice	(Ma et al., 2025)
Remdesivir	o *In vivo*: HFD-induced MASLD mouse model o *In vitro*: PA-treated hepatocytes, STING-overexpressing hepatocyte model	o Directly inhibits STING pathway, blocking IRF3/NF-κB activation, regulating hepatocyte lipid metabolism-related pathways	o Reduces liver weight and lipid accumulation o Improves insulin resistance o Suppresses hepatic systemic inflammation	(Li and Su, 2020)
Phloretin	o *In vivo*: MASH mouse model o *In vitro*: Cell model	o Inhibits pathological mPTP opening via SHP-2/JAK2/BAX axis, preventing mtDNA leakage and suppressing cGAS-STING activation by cytosolic mtDNA.	o Improves hepatic lipid metabolism disorders and inflammation	(Dong et al., 2025)
Chlorogenic acid	o *In vivo*: HFD/MCD-induced MASH mouse model o *In vitro*: Liver sinusoidal endothelial cell (LSEC) model	o Binds TGFβR1 to downregulate ID1, indirectly inhibiting cGAS-STING-mediated inflammatory cell infiltration	o Reverses aberrant liver vascular phenotype o Reduces trans-endothelial migration of inflammatory cells o Alleviates hepatic inflammatory injury	(Ouyang et al., 2025)
Soy isoflavones	o *In vivo*: Gerbil MASLD model	o Downregulates cGAS and STING expression via ERα-cGAS-STING pathway	o Reduces hepatic fat deposition o Improves liver function; suppresses inflammation	(Yang et al., 2025)
ErTao decoction/Naringin	o *In vivo*: CCl_4_-induced mouse liver fibrosis model; STING-deficient mice o *In vitro*: Macrophages and HSCs	o Inhibits STING-mediated macrophage activation and NLRP3 inflammasome o Naringin identified as a potential STING inhibitor	o Reduces collagen deposition o Alleviates liver fibrotic pathology	(Jia et al., 2025)
Honokiol	o *In vivo*: Mouse model of liver injury and fibrosis induced by 20-week high-cholesterol diet. *In vitro*: Human HepG2 cells; mouse BMDMs	o Blocks NLRP3 inflammasome activation, pyroptosis, and cGAS-STING pathway	o Attenuates cholesterol-induced liver injury and fibrosis	(Wang Z. et al., 2025)
huc-MSCs-derived exosomes	o *In vivo*: MASH model induced by 12-week Western diet (high-fat, high-cholesterol, high-fructose) in C57BL/6J mice	o Exosome-enriched miR-24-3p targets macrophage STING gene, promoting M2 polarization	o Attenuates hepatic steatosis, inflammation, and fibrosis; improves metabolism	(Jiang W. et al., 2025)
Ligustilide	o *In vivo*: Mouse liver fibrosis model; Genetic models: Tmem173^−/−^ (STING-KO) and liver-specific STING-KD mice o *In vitro*: Activated HSCs	o Binds G-actin, disrupting cytoskeleton and nuclear membrane integrity, causing YAP leakage and dsDNA release, thereby activating cGAS-STING pathway and inducing HSC senescence	o Ameliorates liver fibrosis; Induces HSC senescence	(Qu et al., 2025)
Oroxylin A	o *In vivo*: Mouse liver fibrosis model o *In vitro*: HSCs (HSC-T6, LX-2)	o Reduces cGAS gene methylation by affecting methionine metabolism, activating cGAS-STING pathway o Activates ferritinophagy via cGAS-STING to induce HSC senescence	o Attenuates liver fibrosis; Induces HSC senescence	(Sun et al., 2023; Zhao et al., 2023)
Atractylenolide III	o *In vitro*: Etoposide-induced senescent human HSC (LX-2) model	o Inhibits cGAS/NF-κB pathway, thereby suppressing senescence-associated secretory phenotype	o Potential anti-fibrotic effect	(Wu et al., 2023)
Manganese-albumin nanosystem	o *In vivo*: Mouse liver fibrosis model	o Delivers Mn^2+^ (cGAS-STING agonist) to activated HSCs via albumin-mediated transcytosis, inducing senescence and enhancing immune clearance	o Limits liver fibrosis	(Gu et al., 2024)

## 5 Limitations and prospects

Despite substantial advances revealing the cGAS-STING pathway’s roles in MASLD progression, multiple critical research gaps remain unresolved. First, cell-type-specific regulatory effects of this pathway are incompletely defined, as the same signaling molecule may exert opposite functions across distinct hepatic cell populations, hindering the development of precise targeted drugs. Second, the stage-dependent bidirectional functions of cGAS-STING across the full spectrum of MASLD have not been systematically characterized, and the threshold between physiological homeostatic activation and pathological overactivation remains poorly defined, with no large multicenter clinical cohorts to validate these effects in humans. Third, the exact molecular targets through which herbal bioactive components modulate cGAS-STING signaling require further verification. Fourth, the reciprocal crosstalk between cGAS-STING signaling and other core pathogenic pathways, such as NLRP3 inflammasome, TGF-β/Smad and NF-κB pathways, in driving MASLD progression remains insufficiently characterized.

All candidate therapeutic agents summarized in this review, including small-molecule monomers, herbal extracts, mesenchymal stem cell-derived exosomes and nano-delivery systems, share consistent preclinical limitations. Most natural compounds possess broad pleiotropic activities, and few studies adopt genetic rescue assays to confirm that their liver-protective effects specifically rely on cGAS-STING regulation. Uniform standardized readouts for pathway activity are absent across laboratories, leading to incomparable experimental outcomes. In addition, biosafety risks, poor *in vivo* stability and hepatic accumulation concerns restrict the clinical translation of nanomedicines and exosome therapies, and no dedicated clinical trials have yet tested cGAS-STING modulators for MASLD/MASH.

No cGAS-STING inhibitor or modulator has received clinical approval for MASLD to date. However, early-phase human trials in autoimmune and oncological diseases provide preliminary proof of human safety and druggability for this pathway class. The oral cGAS inhibitor VENT-03 has completed Phase 1 testing in healthy volunteers with favorable tolerability and clear target engagement (Ventus, 2025), and has advanced to a multicenter, randomized, double-blind, placebo-controlled Phase 2a trial in cutaneous and systemic lupus erythematosus (NCT07260877; ClinicalTrials.gov, 2026). Another oral cGAS inhibitor, IMSB301, finished Phase 1a trials in healthy participants and entered Phase 1b studies for type I interferonopathies such as Aicardi-Goutières syndrome, with the first patient dosed in early 2026 (NCT07364513; ImmuneSensor, 2026).

For oncology indications, multiple STING agonists are undergoing early-phase evaluation in patients with advanced solid tumors: HG381 as monotherapy in a Phase 1 trial (NCT04998422; ClinicalTrials.gov, 2021); TAK-500 as a single agent and in combination with pembrolizumab in a Phase 1/2 trial (NCT05070247; ClinicalTrials.gov, 2023); and TAK-676 (dazostinag) in Phase 1/2 trials both as monotherapy and in combination with pembrolizumab (NCT04420884, NCT04879849; ClinicalTrials.gov, 2020; ClinicalTrials.gov, 2018).

Notably, all these clinical programs target non-metabolic liver indications. Their efficacy, long-term hepatic safety, and optimal dosing in MASLD populations remain completely unvalidated, and dedicated clinical trials for metabolic liver disease are still absent. Existing clinical data cannot be extrapolated to support efficacy or hepatic safety in chronic metabolic liver patients. MASLD poses unique translational obstacles: long-term drug administration is required for patients with comorbid obesity, diabetes and heterogeneous liver fibrosis. Mechanistically, excessive pathway suppression may impair antiviral and anti-tumor immune surveillance, while overactivation drives interferon-mediated hepatic inflammation and systemic cytokine toxicity.

To address the above limitations, future research should advance toward cell-specific, stage-stratified and liver-targeted intervention strategies. Single-cell sequencing and spatial transcriptomics should be prioritized across key cell populations—including hepatocytes, Kupffer cells and liver macrophages, hepatic stellate cells, NK cells, and intestinal epithelial cells—and three critical disease stages. In early steatosis, these tools will define the threshold between homeostatic and pathogenic pathway activation; in progressive fibrosis, they will resolve stage-dependent shifts in immune and stromal cell signaling; in pre-malignant transition, they will characterize the switch from anti-tumor immune surveillance to tumor immune evasion. These approaches will map dynamic cGAS-STING activation profiles across progressive MASLD stages and identify safe, cell-selective therapeutic targets. In line with broader insights from cGAS-STING-targeted drug development, translational efforts must also address core challenges including balancing pathway activation thresholds, avoiding systemic immune suppression, and achieving liver-specific delivery. Optimized liver-targeted delivery carriers, standardized proof-of-mechanism clinical trials, regular surveillance of infection and HCC risk, and in-depth dissection of herbal active ingredient mechanisms will jointly promote the clinical translation of cGAS-STING-targeted precision therapies for MASLD.

## 6 Conclusion

The cGAS-STING pathway, as a central hub of cytosolic DNA sensing and innate immune activation, precisely regulates the full-spectrum progression of MASLD, from simple hepatic steatosis, MASH, and liver fibrosis to HCC, by sensing endogenous stimuli including mtDNA leakage, nuclear DNA damage, and gut microbial DNA, and aberrantly activating downstream signaling axes such as TBK1-IRF3, NF-κB, NLRP3, mTORC1, and YAP. Critically, its regulation exhibits notable cell-type specificity and stage-dependent bidirectionality: moderate physiological activation exerts homeostatic and protective effects, while sustained aberrant overactivation drives pathological progression. It predominantly drives inflammatory amplification and fibrosis in macrophages, regulates lipid metabolism and cell death in hepatocytes, and mediates activation and extracellular matrix deposition in HSCs. Meanwhile, its associated molecules possess biomarker potential for non-invasive MASLD diagnosis and fibrosis staging. Currently, small-molecule compounds, natural products, traditional Chinese medicine monomers, stem cell-derived exosomes, and nanoformulations targeting the cGAS-STING pathway have demonstrated favorable anti-MASLD efficacy in animal models, laying preliminary preclinical groundwork for future clinical exploration.
